# Incidence, treatment, and mortality of ankle fractures: a Danish population-based cohort study

**DOI:** 10.2340/17453674.2025.43006

**Published:** 2025-02-27

**Authors:** Per H GUNDTOFT, Alma B PEDERSEN, Bjarke VIBERG

**Affiliations:** 1 Orthopedic Department, Aarhus University Hospital; 2 Department of Orthopedic Surgery, Kolding Hospital; 3 Department of Clinical Epidemiology, Aarhus University Hospital; 4 Department of Clinical Medicine, Aarhus University; 5 Department of Orthopaedic Surgery and Traumatology, Odense University Hospital; 6 Department of Clinical Research, University of Southern Denmark, Denmark

## Abstract

**Background and purpose:**

Previous studies have shown large variation in the incidence of ankle fractures. Nationwide data covering longer periods is necessary to gain knowledge of the current trends. The aim of this study was to describe the trends in incidence, treatment, and mortality of ankle fracture during a 20-year period.

**Methods:**

Ankle fractures in patients ≥ 18 years old were identified in the Danish National Patient Register using the validated diagnosis and surgical procedure codes for ankle fractures. Incidence rates per 100,000 and incidence rate ratio (IRR) are reported with 95% confidence intervals (CI).

**Results:**

We identified 155,740 ankle fractures. The overall mean incidence rate during the period 1997–2018 was 164 (CI 163–165) per 100,000 person-years, being 154 (CI 152–155) for men and 203 (CI 202–205) for women. The incidence rate increased from 155 (CI 131–179) during 1997–2006 to 173 (CI 147–199) during 2007–2018, corresponding to an IRR of 1.12 (CI 1.10–1.12). This increase was primarily driven by an increase in women, with an IRR of 1.21 (CI 1.20–1.23) and for patients above 50 years, with an IRR of 1.22 (CI 1.08–1.10).

The proportion of patients surgically treated increased from 21% in 1997–2006 to 25% in 2007–2018. The 1-year mortality risk was higher for patients above 65 years with an ankle fracture compared with the general population of the same age, with an IRR of 1.47 (CI 1.42–1.53).

**Conclusion:**

The incidence of ankle fracture increased from 1997 to 2018, primarily due to an increased incidence in women and in the elderly population. The proportion of surgically treated patients increased from 21% to 26%. Excess mortality after ankle fracture in patients above 65 years was observed.

Ankle fractures comprise 10% of all fractures [1,2] making them one of the most common [2,3]. As ankle fractures are often a fragility fracture the incidence is expected to increase as the population continues to age. This predicted increase will be a growing burden for the healthcare system.

Previous studies of the incidence of ankle fractures have shown large variation, with incidence rates between 37 and 187 cases per 100,000 person-years [5-12]. While these studies were performed in different time periods, potential temporal variations alone cannot adequately explain the significant discrepancies observed in incidence rates. The majority of the studies are either single-institute/city based [4,5], or limited to a single region/county [3]. On a national level, studies have reported a wide span of incidences [7-9,12]. These national incidence rates are considerably lower than those reported by non-national studies but are based on unvalidated diagnoses. The large variation in reported incidences could be a result of difference in definition of ankle fractures, data sources used, and low validity. Thus, there is a need for studies on the national level using validated data sources.

The primary aim of our study was to assess the incidence of ankle fractures in relation to age and sex over a 22-year period in Denmark. The secondary aims were to study changes in treatment during the period and the risk of mortality compared with the general population.

## Methods

### Study design

This is a nationwide population-based study using data from the Danish National Patient Register (DNPR) covering the period from 1997 to 2018. The study is reported according to the the REporting of studies Conducted using Observational Routinely-collected health Data (RECORD) guidelines [12].

### Setting

All Danish citizens are guaranteed free access to emergency treatment, general hospital care, and outpatient visit care, as well as primary medical care at public hospitals through the Danish National Health Service [13]. All Danish residents are assigned a unique 10-digit civil registration number at birth or upon immigration. The civil registration number is unchangeable unless the patient has been the victim of identity theft or undertake gender transition. This number is registered in all contacts with the public or private healthcare system and allows for linkage between healthcare registers.

### Data source

The study was based on the DNPR, which has recorded data on all hospital discharges since 1977 [14]. All ankle fractures are treated in public hospitals in Denmark. Reporting to the DNPR is mandatory for both public and private hospitals, whenever patients are using their right to free access to treatment. Therefore, the register capture 99.7% of all hospital discharges in Denmark [14]. The information recorded in the register includes the civil registration number, sex, diagnosis, surgical procedures, and date of birth, hospital admission, and discharge. Diagnoses in the register have been coded according to the Danish Adaption of the 10th revision of the International Classification of Diseases (ICD-10) since 1994. Surgical procedures are coded according to the Nordic Medico-Statistical Committee (NOMESCO) classification system [15].

The Danish Civil Registration System records the civil registration number and provides information on the exact date of death and emigration [16].

### Study population

All patients above 18 years, with a Danish civil registration number and an ankle fracture, were included. We used the following ICD-10 codes for identifying ankle fractures: S82.5, S82.6, S82.7A, S82.7B, S82.8B, and S82.8D. Surgical treatment of an ankle fracture was defined as a relevant surgical procedure performed within 21 days of the diagnosis, as we believed the vast majority of ankle fractures would be treated within that period in Denmark. Non-surgical treatment was defined as the absence of a relevant surgical procedure code within 21 days. The relevant surgical procedure codes were plate (KNHJ60-3), screws alone (KNHJ70-3), nail (KNHJ50-3), wire, rod, cerclage, or pin (KNHJ40-3), external fixation (KNHJ20-3), combined, or other methods (KNHJ80-3, KNHJ90-3). Both the diagnosis code and surgical procedure codes have been validated in a previous study, with the positive predictive value of the ankle fracture diagnosis code of 89% and surgical procedure code of 82% [17].

To estimate the incidence rate we extracted information from Statistics Denmark (Statbank) on the number of Danish citizens of the same age and sex who were alive by the end of each calendar year.

### Variables

Patient age and sex at the date of ankle fracture were determined from the civil registration number. Age was divided into different categories depending on the analysis. For the incidence analysis age was divided into 10-year categories: 18–29, 30–39, 40–49, 50–59, 60–69, 70–79, and ≥ 80. Furthermore, we divided age into 2 age groups, 18–49 and ≥ 50, representing pre- and post-menopause, where the risk of osteoporosis increases. For the mortality incidence estimate, we included only patients aged above 65.

The Charlson Comorbidity Index and the Elixhauser Comorbidity Index are equally good at predicting mortality in an orthopedic setting. Therefore, we decided to report the score for both indices divided into low (0), medium (1–2), and high (> 3) for both scores [18].

### Definition of variables

Surgical treatment was defined as surgery within 21 days of diagnosis. In cases where an external fixation code was followed by another surgical procedure code, e.g., plate osteosynthesis, the case was defined as being treated with the secondary procedure code (KNHJ60) as this was thought to represent cases with temporary external fixation before definitive surgery. However, external fixation can be used as definitive surgical treatment. Since both mono-polar and circular external fixation are reported with the same code, we could not distinguish between the 2.

We only wanted to study first occurrence of an ankle fracture. Therefore, all subsequent readmissions of the same patient and same side were ignored. Furthermore, as a number of reported ankle fractures had missing side, all readmission within 90 days were ignored. This might result in missing some cases, but we believe the risk of bilateral ankle fracture on different dates within 90 days is small and unlikely to influence our results significantly.

### Statistics

We estimated annual overall incidence of ankle fractures as the number of fractures registered in the DNPR by calendar year, divided by the number of individuals of the same age at risk in Denmark. Age- and sex-specific annual incidences were also estimated and expressed per 100,000 individuals in Denmark.

Patient characteristics were described at the time of ankle fracture date using descriptive statistics, with mean values reported including standard deviation (SD). Incidence rate ratio (IRR) was estimated by dividing incidence rates between groups and was estimated as cases per 100,000 person-years, with 95% confidence interval (CI) calculated from the Poisson distribution. The 1-year mortality incidence was estimated only for patients aged above 65 years. All data were analyzed with STATA 17 (StataCorp LLC, College Station, TX, USA).

### Ethics, registration, data sharing plan, funding, use of AI, and disclosures

Data approval was obtained (Region of Southern Denmark, jr.nr. 20/187). According to Danish legislation, no further approval was needed. The authors BV and PHG had full access to all data, which was stored at the Danish Health Data Authority’s research database facilities (“Forskermaskinen”). This study is based on secondary data analysis, so new data is not being measured or produced and all data is available through the DNPR. AI was not used in the writing of this manuscript. The authors received no funding for this work.

None of the authors had any conflict of interest. Complete disclosure of interest forms according to ICMJE are available on the article page, doi: 10.2340/17453674.2025.43006

## Results

Over the 22-year period, 155,740 cases of first-time ankle fractures in 126,789 patients were reported to the DNPR. Of these, the majority were women (57%) and the mean age was 51 years (SD 18), with men being younger than women (mean age 46 [SD 18] vs 56 years [SD 18]) (Table 1). The number of comorbidities was low, with 98% having a Charlson Comorbidity Index score of 0 and 99% having an Elixhauser Comorbidity Index of 0.

**Table 1:** Patient demographics. Values are count and (%) unless otherwise specfied

Factor	Total	1997–2007	2008–2018	P value
Number of cases	155,740	71,522	84,218	
Mean age (CI)	51.9 (51.8–52.0)	49.9 (49.7–50.0)	53.6 (53.5–53.7)	< 0.001
Female sex (%)	(57)	(55)	(60)	< 0.001
Treatment				< 0.001 ^a^
Conservative	118,887 (76)	56,309 (79)	62.578 (74)	
Surgical	36,853 (24)	15,213 (21)	21,640 (26)	< 0.001 ^b^
Plate	25,609 (69)	9,664 (64)	15,945 (74)	
Screw	5,621 (15)	2,931 (19)	2,690 (12)	
Nail	710 (1.9)	111 (0.7)	599 (2.8)	
K-wire	2.404 (6.5)	1,686 (11)	718 (3.3)	
Extern	429 (1.2)	38 (0.2)	391 (1.8)	
Other	2,080 (5.6)	783 (5.1)	1,297 (6.0)	
Charlson Comorbidity Index				< 0.001
Low (0)	126,743 (98)	43,733 (98)	82,970 (99)	
Medium (1–2)	1,883 (1.5)	786 (1.8)	1,097 (1.3)	
High (≥3)	231 (0.2)	80 (0.2)	151 (0.2)	
Elixhauser Comorbidity Index				0.2
Low (0)	127,080 (99)	44,008 (99)	83,072 (99)	
Medium (1–2)	105 (0.1)	29 (0.1)	76 (0.1)	
High (≥3)	1,371 (1.1)	494 (1.1)	877 (1.0)	

CI = 95% confidence interval.

a Conservative versus surgical;

b Distribution of surgical methods

**Figure 1:**
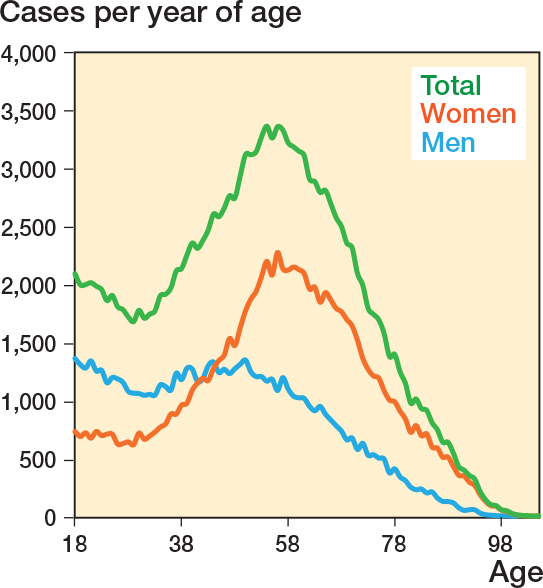
Absolute number of ankle fractures cases per sex and total.

The number of fractures per age showed a bi-apex distribution (Figure 1). For women it peaked at age 55, while men had a plateau from age 18 to 50 followed by a slow decrease. The most common fracture was of the lateral malleolus (Table 2).

**Table 2:** Type of diagnosis for patients with ankle fractures. Values are count and (%)

Fracture site	ICD-10	Overall	Male	Female	P value	Age ≤ 65	Age > 65	P value
Medial malleolus	DS825	26,013 (17)	12,726 (19)	13,287 (15)	< 0.001	19,661 (17)	6,352 (16)	< 0.001
Lateral malleolus	DS826	110,935 (71)	46,849 (71)	64,086 (72)	< 0.001	83,522 (73)	27,413 (67)	< 0.001
Bimalleolar	DS827A	5,002 (3.2)	1,659 (2.5)	3,343 (3.7)	< 0.001	2,870 (2.5)	2,132 (5.2)	< 0.001
Trimalleolar	DS827B	4,976 (3.2)	1,524 (2.3)	3,452 (3.9)	< 0.001	3,126 (2.7)	1,850 (4.5)	< 0.001
Unspecified	DS828B+D	8,814 (5.7)	3,449 (5.2)	5,365 (6,0)	< 0.001	5,865 (5.1)	2,949 (7.2)	< 0.001
Total		155,740	66,207	89,533		115,044	40,696	

### Incidence

The overall mean incidence was 164 (CI 163–165) per 100,000 person-years during the entire period from 1997 to 2018. Women had an overall incidence of 203 (CI 202–205), while this was 154 (CI 152–155) for men. This corresponds to an IRR of 1.32 (CI 1.31–1.34) for women compared with men.

**Figure 2:**
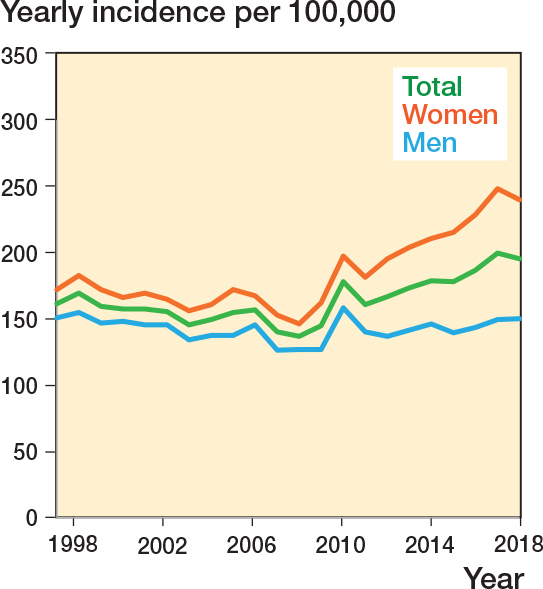
Ankle fracture incidence over time stratified by sex.

**Figure 3:**
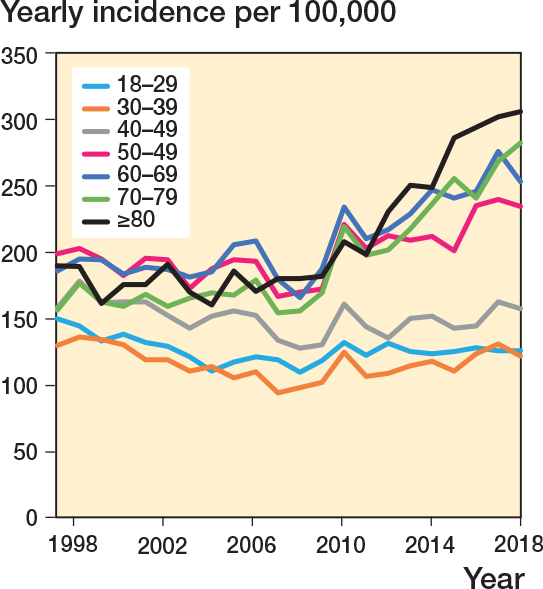
Ankle fracture incidence over time stratified by 10-year age increments.

In 1997–2007 the incidence rate was 155 (CI 126–184) cases per 100,000, which increased to 173 (CI 145–201) in 2008–2018. This corresponds to an IRR of 1.12 (CI 1.10–1.13) for the later period compared with the earlier. This increase was primarily due to an increased incidence in women (Figure 2) and predominantly seen in patients aged above 50 (IRR 1.22 [CI 1.08–1.10] for the late vs early period) (Figure 3). For patients below the age of 50 the corresponding IRR was 0.96 (CI 0.95–0.97).

### Treatment

**Figure 4:**
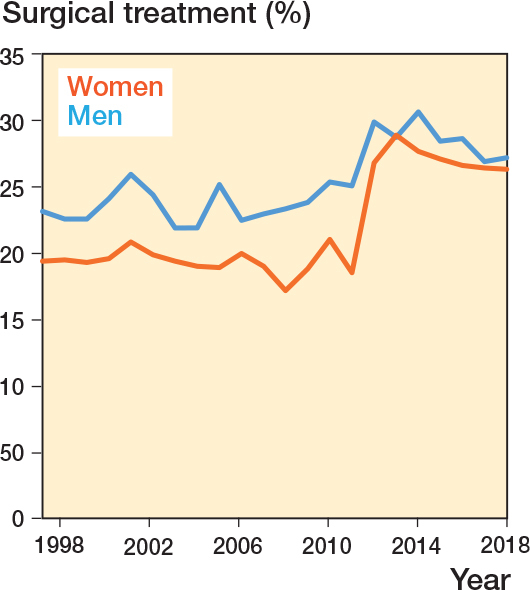
Surgical treatment over time stratified by sex.

Most ankle fractures were treated non-surgically (76%), but during the later period the percentage of surgically treated ankle fractures increased from 21% to 26% (P < 0.001). Surgical treatment was dominated by plate osteosynthesis (69%). Men were surgically treated in 25% of cases, which was higher than in women (22%) (P < 0.001). The percentage of surgical treatment increased for both men and women, but with a greater increase for women, resulting in a similar percentage of surgically treated fractures for men and women during the later period (Figure 4).

### Mortality

Patients above 65 years of age had a higher 1-year mortality incidence than the general Danish population above 65 (7,369 and 5,003 per 100,000 per person-years, IRR 1.47 [CI 1.42–1.53]). This increased mortality risk was consistent for all age groups above 65, with an IRR of 1.43 (CI 1.35–1.52) for those aged 65–79; 1.47 (CI 1.38–1.56) for 80–89; and 1.26 (CI 1.15–1.37) for > 90 years.

## Discussion

The primary aim of our study was to assess the incidence of ankle fractures in relation to age and sex over a 22-year period in Denmark. During this period, we observed an increase in the incidence of ankle fractures overall and an increase in surgical treatment. The increasing incidence was primarily due to an increasing risk of ankle fractures for women and for patients aged above 50. The increase in surgical treatment was most pronounced for women, resulting in the same rate of surgery for both sexes during the later period. Moreover, we showed that patients aged above 65 years had a higher 1-year mortality risk than the general Danish population.

The overall incidence of 164 per 100,000 is higher than reported by other national studies: 1 study from the USA reported an incidence of 42 per 100,000 and 2 from Sweden reported incidences rates of 71 and 152 per 100,000 [6,8]. Here, the Swedish studies are most comparable to ours: Thur et al. included only individuals with ankle fractures admitted to hospital, in contrast to our inclusion of all patients [6]. As the majority of patients with ankle fractures are treated as outpatients in the emergency room, this can to a large extent explain our higher incidence. Rydberg et al. reported a slightly lower incidence than ours by using data from the Swedish Fracture Register [11]. The completeness of ankle fracture registrations of 70% in this register could explain the difference. A study including only the northern region of Denmark found an incidence of 169 per 100,000, similar to our result [19]. Therefore, we believe that the incidence of 164 per 100,000 person-years is very close to the true incidence of ankle fractures.

Our finding of increasing incidence supports earlier studies from the Netherlands and Germany [20,21], but is contradicted by the regional Danish study [19]. We found the increase to be primarily caused by an increasing incidence for women and for patients aged above 50 years. It is evident that women’s risk increases with age in contrast to men, presumably due to the risk of fragility fractures. Patients in the later period were older, thereby incurring an increasing risk of osteoporosis. This could to some extent explain the increasing incidence, although the reason for women’s and older patients’ increasing risk in the later period is possible multifactorial; for example, increasing participation in sports and other recreational activities, as studies have shown that an increasing number of ankle injuries are due to sport [22].

The percentage of surgical treatment increased from 21% to 26%, due to more women having surgery in the later period. From 2012 onward the surgical treatment of women was similar to that for men, which is in contrast to a study by Ranganathan et al., who showed that in the USA women with displaced ankle fractures were less likely than men to receive surgical treatment in the period 2004 to 2021 [23]. The reason for this difference between men and women could be due to both surgeons and patient factors, but somehow this changed in Denmark during the study period, as the distribution of non-surgical and surgical treatment was similar for women and men in the last period. The increase in surgical treatment has not been studied previously for ankle fractures, but a similar increase in surgical treatment has been shown for the distal radius in Denmark [24]. There are no national guidelines or meta-analyses in the period that can explain this increase [25]. A possible explanation for the increase in surgical treatment could be that patients are becoming more active, in line with the increasing number of ankle injuries being a result of sport, or that surgeons have become more inclined to treat ankle fractures with surgery [22,26]. Following the end of the study period new diagnostic tests, including weight-bearing radiographs, and studies on non-surgical vs surgical treatment demonstrated that in numerous cases non-surgical treatment is non-inferior to surgery [4,27-30]. Consequently, the proportion of patients undergoing surgical treatment may decline in the period following this study.

### Strengths

The strength of this study is that it is based on validated data from a large nationwide population covering more than 2 decades. Furthermore, as all ankle fractures are treated at a public hospital in Denmark, this study has complete nationwide coverage. Therefore, the external validity of the study is high.

### Limitations

Using national patient registers runs the risk of overestimating the incidence due to registration of readmissions [31]. To reduce this inaccuracy caused by readmission we established a 90-day “quarantine period.” A second limitation is that, although all ankle fractures, including those diagnosed by general practitioners, are referred to and managed at public hospitals, some patients with ankle fractures may mistakenly not be registered in the DNPR by hospitals. Further, some patients may not seek medical attention and are therefore not registered in the DNPR. A third limitation is that we were not able to adjust for calendar year, age, sex, and comorbidities, when comparing the mortality incidence for patients above 65 years of age with ankle fractures and the same-age general population. Therefore, the association between ankle fractures and higher mortality incidence might be due to a high number of comorbidities in patients with ankle fractures. The increased mortality for patients with ankle fractures could be caused by other risk factors, e.g., immobilizations, as is seen in patients with hip fractures.

### Conclusion

The incidence of ankle fracture increased from 1997 to 2018, primarily due to an increased incidence in women and in the elderly population. Contemporary with this increase the proportion of surgically treated patients increased from 21% to 26%. Excess mortality after ankle fracture in patients aged above 65 years was observed.

## Notes

PHG: conceptualization of the study, conducting data analysis, creating tables and figures, drafting the initial manuscript, and editing the manuscript following review by BV and ABP. ABP: contributed to the study design, data analysis and interpretation of the results, and critical revision of the manuscript. BV: obtaining access to data, funding, conceptualization of the study, data analysis, and critical revision of the manuscript.

Handling co-editors: Cecilia Rogmark and Philippe Wagner

*Acta* thanks Marius Molund and Emilia Möller Rydberg for help with peer review of this manuscript.
